# Phase 2 study of vismodegib, a hedgehog inhibitor, combined with gemcitabine and nab-paclitaxel in patients with untreated metastatic pancreatic adenocarcinoma

**DOI:** 10.1038/s41416-019-0683-3

**Published:** 2019-12-20

**Authors:** Ana De Jesus-Acosta, Elizabeth A. Sugar, Peter J. O’Dwyer, Ramesh K. Ramanathan, Daniel D. Von Hoff, Zeshaan Rasheed, Lei Zheng, Asma Begum, Robert Anders, Anirban Maitra, Florencia McAllister, N. V. Rajeshkumar, Shinichi Yabuuchi, Roeland F. de Wilde, Bhavina Batukbhai, Ismet Sahin, Daniel A. Laheru

**Affiliations:** 1 Department of Medical Oncology, Kimmel Comprehensive Cancer Center at Johns Hopkins Hospital, Baltimore, MD USA; 2 Department of Biostatistics, the Johns Hopkins Bloomberg School of Public Health, Baltimore, MD USA; 3 Abramson Cancer Center, University of Pennsylvania, Philadelphia, PA USA; 4 Honor Health Research Institute & Translational Genomics Research Institute, Scottsdale, AZ USA; 5 Department of Oncology, The Sidney Kimmel Comprehensive Cancer Center, Johns Hopkins University School of Medicine, Baltimore, MD USA; 6 Departments of Pathology, The Sidney Kimmel Comprehensive Cancer Center, Johns Hopkins University School of Medicine, Baltimore, MD USA; 7 Departments of Pathology, The University of Texas MD Anderson Cancer Center, Houston, TX USA; 8 Department of Clinical Cancer Prevention, The University of Texas MD Anderson Cancer Center, Houston, TX USA; 9 Department of Surgery, Sendai South Hospital, Sendai, Japan; 10 Department of Engineering, Texas Southern University, Houston, TX USA

**Keywords:** Cancer therapy, Gastrointestinal cancer

## Abstract

**Background:**

The Hedgehog (Hh) signalling pathway is overexpressed in pancreatic ductal adenocarcinoma (PDA). Preclinical studies have shown that Hh inhibitors reduce pancreatic cancer stem cells (pCSC), stroma and Hh signalling.

**Methods:**

Patients with previously untreated metastatic PDA were treated with gemcitabine and nab-paclitaxel. Vismodegib was added starting on the second cycle. The primary endpoint was progression-free survival (PFS) as compared with historical controls. Tumour biopsies to assess pCSC, stroma and Hh signalling were obtained before treatment and after cycle 1 (gemcitabine and nab-paclitaxel) or after cycle 2 (gemcitabine and nab-paclitaxel plus vismodegib).

**Results:**

Seventy-one patients were enrolled. Median PFS and overall survival (OS) were 5.42 months (95% confidence interval [CI]: 4.37–6.97) and 9.79 months (95% CI: 7.85–10.97), respectively. Of the 67 patients evaluable for response, 27 (40%) had a response: 26 (38.8%) partial responses and 1 complete response. In the tumour samples, there were no significant changes in ALDH + pCSC following treatment.

**Conclusions:**

Adding vismodegib to chemotherapy did not improve efficacy as compared with historical rates observed with chemotherapy alone in patients with newly diagnosed metastatic pancreatic cancer. This study does not support the further evaluation of Hh inhibitors in this patient population.

**Trial registration:**

ClinicalTrials.gov Identifier: NCT01088815.

## Background

Pancreatic cancer is one of the most deadly malignancies in the gastrointestinal tract and remains the fourth leading cause of cancer-related mortality. In the United States, an estimated total of 55,440 pancreatic cancer diagnoses are expected to occur in 2018 with 44,330 expected deaths.^1,2^ Standard regimens for patients with metastatic pancreatic ductal adenocarcinoma (PDA) include single agent gemcitabine or combination regimens, such as gemcitabine with nab-paclitaxel^3^ and the FOLFIRINOX^4^ regimen. Patients treated with these regimens have an estimated survival ranging from 6 to 11 months, depending upon the therapy. Several signalling pathways have been shown to play a role in cancer development and progression, and are being investigated as a channel for improving outcomes for this disease.

The Sonic Hedgehog (Hh) signalling pathway regulates epithelial and mesenchymal interactions in a variety of tissues during mammalian embryogenesis.^5^ The Hh ligand in the extracellular space binds to Patched (PTCH), a 12-pass transmembrane receptor on the surface of cells. Hh binding relieves the inhibitory effect of PTCH on Smoothened (SMO), a 7-pass transmembrane domain protein and a member of the G-protein-coupled receptor superfamily. Signal transduction by SMO then leads to the activation and nuclear localisation of GLI-1 transcription factors and induction of Hh target genes, many of which are involved in proliferation, survival and angiogenesis in different malignancies.

In PDA, the Hh signalling pathway has been reported to be critical for tumour progression in preclinical pancreatic cancer models.^6,7^ This pathway is persistently activated^7,8^ either through aberrant ligand overexpression by pancreatic cancer cells^8^ or aberrant activation in the surrounding stromal compartment.^8–10^ When activated, it promotes epithelial–mesenchymal transition and increases motility and invasiveness of pancreatic cancer cells. Using clinical samples, studies have shown that Hh activation is associated with poor prognosis in patients with PDA.^11,12^ This pathway has also been shown to sustain pancreatic cancer stem cells (pCSCs) that comprise a small fraction of pancreatic cancer cells (0.2–0.8%), but are highly tumorigenic and possess the ability of self-renewal and produce a differentiated progeny.^12–15^ pCSC has shown chemotherapy drug resistance and a role in metastatic disease progression.^12,13,16^

Based on the above findings, there was increased interest in the development of therapeutic strategies capable of downregulating the Hh pathway in an effort to eliminate pCSCs and the surrounding stroma. Preclinical studies showed that combining Hh inhibitors with chemotherapy improved outcomes in animal models of PDA.^8,17–19^ We conducted a clinical trial evaluating the safety and efficacy of the Hh inhibitor vismodegib (GDC-0449) in combination with chemotherapy in patients with metastatic PDA. The trial included correlative studies aimed to identify the effect of Hh pathway inhibition in pancreas CSC and tumour stroma.

## Methods

This is an open-label Phase 2 clinical trial evaluating the safety and efficacy of the Hh inhibitor, vismodegib (GDC-0449), in combination with chemotherapy gemcitabine and nab-paclitaxel in patients with previously untreated metastatic PDA. Patients were enrolled at three different institutions: the Johns Hopkins Kimmel Comprehensive Cancer Center in Baltimore, MD; the Abramson Cancer Center of the University of PA in Philadelphia, PA and the Translational Genomics Institute in Scottsdale, AZ. The study was approved by the Institutional Review Boards at each institution, and all participants provided signed informed consent. This study was conducted in accordance with the Declaration of Helsinki for Good Clinical Practice guidelines. For additional information on approval numbers, see the section ‘Ethics approval and consent to participate'.

### Study treatment and eligibility

Patients were eligible if they had newly diagnosed metastatic PDA without previous treatment or with adjuvant therapy completed > 6 months prior to diagnosis of metastatic disease. Other eligibility criteria included measurable disease based on independent review by a radiologist using RECIST v1.1 criteria, Eastern Cooperative Oncology Group (ECOG) performance score of 0–1, adequate bone marrow, renal and hepatic function. Therapy consisted of one cycle of gemcitabine 1000 mg/m^2^ and nab-paclitaxel 125 mg/m^2^ on days 1, 8 and 15 for the first 28-day cycle followed by gemcitabine 1000 mg/m^2^, nab-paclitaxel 125 mg/m^2^ on days 1, 8 and 15 every 28 days in combination with oral vismodegib (GDC-0449) 150 mg daily for subsequent cycles. Vismodegib at the dose of 150 daily is the recommended Phase 2 dose based on Phase 1 clinical trials.^20^ Treatment was continued until evidence of disease progression or unacceptable toxicities. All imaging studies and responses were assessed independently by certified radiologists using RECIST criteria guidelines. Patients were followed every 6 months for overall survival for a total of 18 months.

### Correlative studies

We evaluated two sources of patient material (tumour core biopsies and blood). The pre-treatment biopsies and blood samples for circulating stem cells were obtained before initiating treatment. The second biopsy and blood samples alternated between the first or second cycles based on assigned participant ID number at study enrolment. This allowed for the evaluation of both the effect due to chemotherapy only (after cycle 1) and with chemotherapy plus vismodegib (after cycle 2).

#### Tumour biopsies

Core biopsies were obtained using a 22-gauge needle to limit procedure-related complications. Unstained 5 -µm thick ChemMate slides were cut for correlative studies. Tumour biopsies were performed in the primary pancreas tumour or an accessible metastatic lesion.

#### Aldehyde dehydrogenase (ALDH) immunohistochemistry (IHC)

We used ALDH as our marker for pCSC and used IHC for detecting its expression. This IHC marker has been previously validated by our group.^14^ Paraffin-embedded biopsy sections were deparaffinised in xylene and rehydrated in graded alcohol washes. Deparaffinised sections were immersed in boiling sodium citrate buffer (10 mM, pH 6.0) for 30 min. Endogenous peroxidase activities were quenched with 3% hydrogen peroxide in methanol for 10 min at room temperature. Sections were washed and incubated with a mouse monoclonal antibody against human ALDH-1 (clone 44; BD Biosciences) diluted 1:50 at room temperature for 1 h followed by antibody detection with the use of a Vectastain Elite developer kit (Vector Labs) and 3,3′-diamniobenzidine. A pathologist blinded to the results of patients’ clinical outcomes reviewed each tumour sample. A tumour area was identified and selected for IHC staining evaluation with the inclusion of neoplastic tissue. IHC was graded as negative (0) if no staining evident, weak positive or strong positive.

#### Gli-1 immunohistochemistry

We used Gli-1 expression as a biomarker for Hh signalling activation. Gli-1 staining was validated by our immunopathology core. Formalin-fixed paraffin-embedded slides of tissue were deparaffinised and hydrated. Heat-mediated antigen retrieval was performed using EDTA, pH 8 buffer by heating the slides under pressure at 125 °C for 30 s followed by 95 °C for 10 s. Following manual antigen retrieval, slides were stained using a standard protocol on a Leica Microsystems Bond auto-staining machine. Following Snipr treatment (Leica Microsystems), the primary antibody (Santa Cruz) was incubated for 30 min at a 1:200 dilution. The slides were then incubated with a secondary antibody from the bond polymer REFINE detection kit (Leica Microsystems). Development was performed using 3,3′-diaminobenzidine hydrochloride (DAB), and the slides were counterstained with haematoxylin. Slides were digitally scanned. A pathologist blinded to the results of patients’ clinical outcomes reviewed each tumour sample. A tumour area and when available a stromal area was identified and selected for grading. Staining was graded as negative (0) if no staining was evident, positive cytoplasmic staining or positive with both nuclear/cytoplasmic staining. Tumour differentiation was graded as well, moderately or poorly differentiated tumours.

#### Immunofluorescent labelling

We used actin α-smooth muscle (SMA) as a marker of myofibroblasts, the main component of the PDA desmoplastic stroma.^21^ CD45 was our biomarker for immune cell infiltration.^22^ After fixation and paraffin embedding, tissue was cut into 5-μm sections. Antigen retrieval was performed by placing slides containing the sections in a citrate-based antigen-unmasking solution (Vector Laboratories) and boiling them in a microwave. Sections were permeabilised for 30 min in 0.2% Triton X-100 in TBS, and blocking of nonspecific reactivity was performed for 45 min in 10% FBS/0.2% Triton X-100 in TBS at room temperature (RT). Anti-CD45 (BD Biosciences) primary antibody was diluted (1:100) in 5% FBS/0.2% Triton X-100/TBS  and incubated overnight. Slides were washed three times in 0.2% Triton X-100 in PBS, and sections incubated with Alexa-647-conjugated secondary antibodies for 1 h at RT in the dark. Alexa 488-conjugated anti-E-cadherin (BD Biosciences) antibody was used at a concentration of 1:100. Cy3-conjugated anti-actin α-smooth muscle (SMA; SIGMA) was used at 1:750. Nuclei were labelled with DAPI (1:1000), and slides were mounted in Vectashield mounting medium (Vector Labs). Images were acquired using Nikon confocal imaging.

#### Immunofluorescence image analysis

Images were processed using NIS Elements software (Nikon) and analysed with Image J software. Images were analysed by quantifying surface area occupied by stained cells (E-cadherin/SMA) and expressed as percentage of the total surface area. CD45 + cells were individually quantified, and their number expressed as percentage of total cells in the field. To calculate the total number of cells, each image was passed through a median filter, and the smoothed image was converted to a binary image based on the autothreshold calculated with the ‘IsoData dark' method in Image J. The watershed algorithm was applied to the binary image to obtain the segmented number of DAPI + cells.

#### Cell staining and flow cytometry

Peripheral blood mononuclear cells (PBMC) were stained with ALDEFLUOR reagent (Stem Cell Technologies) for 30 min in a 37 °C water bath according to the manufacturer’s protocol. Cells were washed and subsequently stained for 30 min on ice with a mix of anti-human EpCAM-PerCP/cy5.5 antibody (Abcam, Cambridge, UK), anti-human CD45 microbeads (Miltenyi Biotec, Bergisch Gladbach, Germany), anti-human biotin-conjugated CD31 antibody (Invitrogen, Carlsbad, CA), anti-biotin microbeads (Miltenyi Biotec), anti-human CD45-APC antibody (BD Biosciences) and anti-APC streptavidin antibodies (BD Biosciences). Cells were washed twice with 1× aldefluor assay buffer. Leucocytes (CD45 + cells) and endothelial cells (CD31 + cells) were depleted using LD columns and MACS Separator (Miltenyi Biotec). BD LSR II (BD Biosciences) was used for cell analysis. Anti-human CD45-APC and anti-APC streptavidin antibodies were used as a precaution to detect and discard residual leucocytes or endothelial cells by FACs.

#### RNA extraction, cDNA synthesis and quantitative real-time PCR (qRT-PCR)

The total RNA was extracted from the pre- and post-biopsy samples using the RNeasy PlusMini Kit (Cat. No. 74106, Qiagen), and reverse-transcribed with SuperScript III reverse transcriptase (Cat. No. 4368814, Invitrogen). qRT-PCR was carried out using TaqMan probes (Applied Biosystems) against human *ACTB* (Hs99999903_m1), *GLI-1* (Hs01110766_m1) and *PTCH-1* (Hs00181117_m1). qRT-PCR was performed in triplicate for each sample. The relative mRNA expression of *GLI-1* and PTCH1 was normalised to internal control *ACTB*, and estimated using the 2^−ΔΔ*Ct*^ method.^23^

### Statistical analysis

The primary objective was to evaluate whether the addition of a Hh inhibitor (GDC-0449) to cytotoxic chemotherapy (gemcitabine and nab-paclitaxel) increased progression-free survival (PFS) in patients with metastatic PDA. Secondary objectives included evaluating the overall survival (OS), tumour response, correlative endpoints and the safety of the combination gemcitabine + nab-paclitaxel with GDC-0449 in patients with metastatic PDA.

The original sample size was 80 patients, which would have provided 80% power to detect an increase in the median PFS from the reference of 8 months (based upon historical control data available at the time of study design from Phase 1/2 clinical trial of gemcitabine + nab-paclitaxel)^24^ to 10.4 months, a 30% increase, with a one-sided type I error of 0.10. The sample size was modified based upon the results of the IMPACT trial, a Phase 3 study in which the median PFS for gemcitabine + nab-paclitaxel was 5.5 months.^3^ A reduced sample size of 72 provided 80% power to detect a 30% increase from the new reference of 5.5–7.15 months assuming a one-sided type I error of 0.10.

PFS was defined as the time from treatment initiation to the earlier documented disease progression or death from any cause. Overall survival was calculated from treatment initiation until death from any cause. For both outcomes, individuals without an event were censored at the date of last contact. Kaplan–Meier estimates of the survival function were used to graphically display time-to-event outcomes (PFS, OS) as well as to calculate the median and cumulative proportion at 1 year with 95% confidence intervals. Log-rank tests and Cox proportional hazards models were used to assess the impact of risk factors on time-to-event outcomes. Risk factors included age (> 65 vs. ≤ 65), gender (female vs. male), race (white, black, Asian), prior resection (no vs. yes), ECOG status (0 vs. 1), nodal status (negative vs. positive) and the standard definition of CA 19-9 elevation (< 37 vs. ≥ 37), as well as a more extreme threshold (< 5000 vs. ≥ 5000).

Objective tumour response (partial or complete response) and stable disease were evaluated according to RECIST 1.1 criteria by an independent radiology review.^25^ All patients were included in the analysis as intention to treat. The proportion of individuals with a response by the end of four cycles was computed. Individuals who died, progressed or were lost to follow-up prior to this time point were counted as nonresponders. Fisher’s exact tests and Wilcoxon rank-sum tests were used to assess the impact of clinical and demographic characteristics on response.

Toxicities were graded according to National Cancer Institute Common Toxicity Criteria for Adverse Events (NCI CTCAE, Version 4.0). To evaluate safety, the number, proportion and incidence (events per cycle) of toxicities that occurred after treatment initiation and the number of affected individuals were calculated for all toxicities as well as for the subset of SAEs. Analyses was repeated for those toxicities that were designated as definitely, probably or possibly related to study medication. A detailed listing of the type, timing and severity of each SAE is also provided. Biomarker measurements were taken at baseline (pretreatment) as well as post treatment (either post chemotherapy or post vismodegib). For tumour biopsies, ALDH was graded as negative (0) if no staining was evident, weak positive or strong positive. GLI-1 staining was graded in tumour and stroma as negative (0) if no staining was evident, positive cytoplasmic staining or positive with both nuclear/cytoplasmic staining. Tumour differentiation was graded as well-differentiated, moderately differentiated or poorly differentiated tumours. For paired biopsy measurements, the number remained stable, improved or worsened, which was tabulated. For circulating tumour cells, the median and 1st to 3rd quartile was calculated for each time point, as well as the ratio of the post-treatment divided by pre-treatment measurements. Wilcoxon rank-sum and signed-rank tests were used to compare unpaired and paired measurements of circulating tumour cells, respectively. Baseline measurements of circulating tumour cells were dichotomised based upon the median (i.e. < median vs. ≥ median). The impact of baseline biopsy and circulating tumour cells on PFS and OS was assessed using the techniques described above.

All statistical analyses were performed using R (The R Project for Statistical Computing, http://www.r-project.org/). *P* values were considered significant if <0.05.

## Results

### Patients and treatment administration

A total of 95 patients were screened (Supplementary Fig. 1). Twenty four were excluded due to screening failures (*N* = 19) or withdrawing consent prior to initiation of treatment (*N* = 5). A total of 71 participants had follow-up, and demographics summarised in Table 1. A total of seven (10%) did not start the vismodegib (GDC-0449): one died, two had progressive disease, one had an SAE with declining performance status and the other three were precluded due to illness or infection. The median number of cycles completed was four (range: 1–14). The reasons for coming off study treatment included progression (*N* = 35, 49%), toxicity or complications (*N* = 26, 37%), death (*N* = 3, 4%), patient withdrawal (*N* = 2, 3%) or other reasons (*N* = 5, 7%).

**Table 1:** Demographic and disease characteristics of participating subjects.

Characteristic	Summary
*Demographics*	
Age at enrolment, median (Q1–Q3)	62 (57–70)
Male, N (%)	37 (52%)
Race^a^	
White	63 (90%)
Black	4 (6%)
Asian	3 (4%)
*Disease status*	
ECOG at enrolment, N (%)	
0	30 (42%)
1	41 (58%)
Current disease involvement, N (%)^b^	
Primary tumour	60 (87%)
Liver	59 (86%)
Regional lymph nodes	26 (38%)
Lung	17 (25%)
Distant lymph nodes	15 (22%)
Peritoneum	12 (17%)
CA 19-9 at enrolment^c^	
Median (Q1–Q3)	1500 (123, 7260)
Elevated (≥ 37), *N* (%)	53 (84%)
Prior surgical resection, *N* (%)^d^	7 (10%)

*Q1*  1st quartile, *Q3*  3rd quartile, N   number, *%*   percent

^a^Race is unknown for one individual

^b^The total is greater than 100% since multiple locations are possible. Two individuals were missing disease involvement

^c^Baseline CA 19-9 is missing for eight individuals

^d^Two patients are missing data on prior resection

### Efficacy

**Fig. 1:**
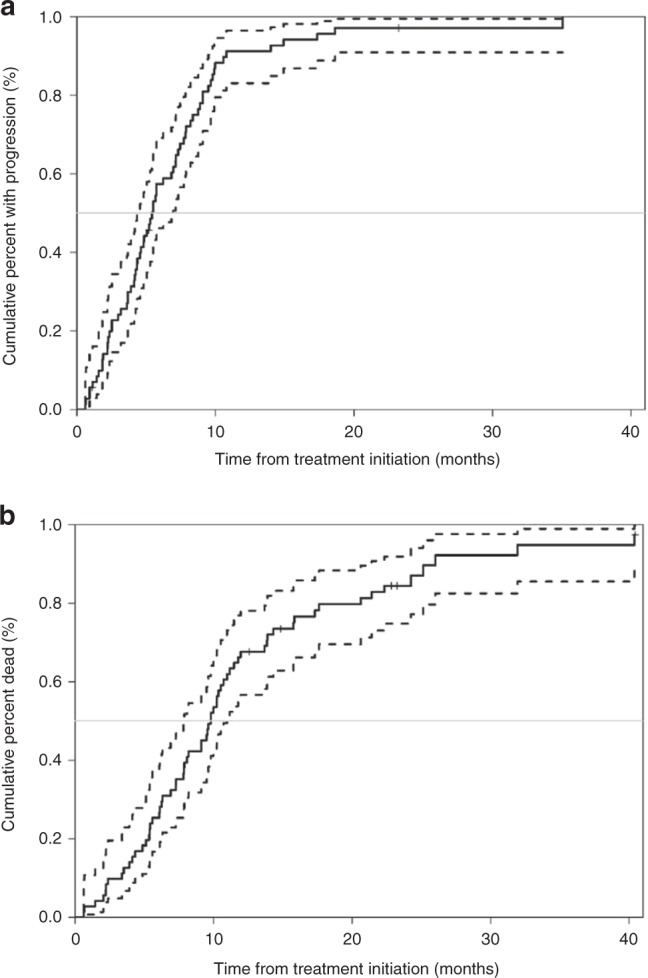
Kaplan–Meier estimates of survival. The estimate is represented with a solid line and the 95% confidence interval is represented with dotted lines. Kaplan–Meier estimates of (**a**) progression-free survival and (**b**) overall survival. The estimate is represented with a solid line and the 95% confidence interval is represented with dotted lines.

At the time of analysis, 64 individuals died, and 68 individuals progressed during follow-up. The median time to progression was 5.42 months (95% confidence interval [CI]: 4.37–6.97, Fig. 1a). The median time to death was 9.79 months (95% CI: 7.85–10.97, Fig. 1b). Although simply having an elevated (defined as ≥ 37) CA 19-9 at baseline was not associated with either progression or OS, individuals with a baseline CA 19-9 ≥ 5000 had a significantly shorter time to progression (HR = 2.8, 95% CI: 1.56–5.03, *p* = 0.0005) and death (HR = 3.15, 95% CI: 1.74, 5.70, *p* = 0.0001) than those with a baseline CA 19-9 < 5000. Individuals with ECOG status of 1 had OS that was borderline significantly shorter than those with excellent performance ECOG 0 (HR 1.56, 95% CI: 0.93, 2.61, *p* = 0.09); however, the impact was attenuated and was not statistically significant for the primary outcome PFS (Supplementary Tables 1, 2).

A total of 67 patients were evaluable for response, and 4 were not. Of these, 27 (40%) had a response: 26 (38.8%) partial responses [PR] and 1 complete response [CR]. There was no significant association between any of the baseline demographics, disease characteristics or correlative markers and response status after four cycles (Supplementary Table 3).

### Adverse events

A total of 104 SAEs were observed (Table 2). Of these, 18 SAEs among 8 individuals were considered related to study treatment. Only 6 of the 18 related SAEs occurred while taking Vismodegib although not specifically attributed to vismodegib (Table 3). Supplementary Table 4 summarises the AEs reported with single agent vismodegib on previous Phase 1 trial and the frequency to which these AEs occurred in our trial.

**Table 2:** Adverse events observed during follow-up.

Category	Overall number of AEs (number of individuals)	Rate of AEs: AEs/number of cycles
*All toxicities*		
SAEs	104 (46)	0.29
Grade		
5	5 (5)	0.014
4	4 (45)	0.011
3	436 (64)	1.20
2	876 (70)	2.42
1	1732 (71)	4.78
*Toxicities related to study medication*^a^		
SAEs	18 (8)	0.05
Grade		
5	0	0
4	0	0
3	42 (19)	0.12
2	91 (32)	0.25
1	166 (47)	0.46

^a^Designated as definitely, probably or possibly related to any of the study medications

**Table 3:** Detailed listing of the type and severity of SAEs designated as being possibly, probably or definitely related to study treatment.

Toxicity	Observed while taking vismodegib	Maximum grade
Pancytopenia^a^	Yes	3
Febrile neutropenia^b^	Yes	3
Neutrophil count decreased^c^	Yes	3
Platelet count decreased^c^	Yes	3
Pneumonitis^b^	Yes	3
Pulmonary drug toxicity	Yes	3
Dehydration	No	3
Fever^a^	No	2
Pneumonitis	No	3
Sepsis	No	3
Anaemia^d^	No	3
Dehydration^d^	No	3
Fatigue^d^	No	2
Liver abscess^d^	No	3
Nausea^d^	No	3
Sepsis^d^	No	3
Vomiting^d^	No	3
General disorders/administration site condition^d^	No	2

^a,^^b,^^c,^^d^Toxicities observed on the same individual

None of these were suspected related to vismodegib

### Correlative studies

Of the 71 patients treated in the study, 66 (93%) provided pre-treatment core biopsies and 51 (72%) provided post-treatment core biopsies for correlates. Upon histologic assessment of collected specimens, there was adequate amount of tumour for analysis in 49/66 (74%) pre-treatment biopsies and 35/51 (69%) of the post-treatment biopsies. Supplementary Fig. 2 delineates sample acquisition.

### IHC staining for ALDH

IHC staining for ALDH assessment was available for 40 pre-treatment biopsies and 23 paired post-treatment biopsies. Of these 23 paired samples, 20 had no change from pre- to post-treatment biopsy, 2 changed to less intense and 1 changed to more intense. Baseline tumour measurements of ALDH were not significantly associated with PFS or OS (*p* > 0.05, Supplementary Tables 1, 2). No comparisons between the patients that had their second biopsy post chemotherapy or post Hh were made as the majority of the patients (20/23) had no change from baseline.

#### Flow cytometry for circulating pancreatic cancer stem cells

Flow cytometry for ALDH-bright cells was performed on PBMC samples from 57 individuals pretreatment. Of these, 11 had matching post-chemotherapy samples, 33 had matching post-vismodegib samples and 13 did not have matching follow-up circulating tumour data (Table 4). There were no significant changes in ALDH + , ALDH + EpCAM + either post chemotherapy or post vismodegib. EpCAM + was increased after chemotherapy (*p* = 0.037), but not after vismodegib. None of the baseline circulating tumour cell measurements were significantly associated with PFS or OS (*p* > 0.05, Supplementary Tables 1, 2).

**Table 4:** Biomarkers from circulating tumour cells taken prior to treatment, after chemotherapy initiation and after vismodegib initiation.

	Pre-treatment	Post chemotherapy	Ratio expression: post chemotherapy/pre-treatment	Post vismodegib	Ratio of expression: post vismodegib/pre-treatment
	*N* = 57	*N* = 12	*N* = 11	*N* = 34	*N* = 33
ALDH + ^a^	27.0 (4.0, 168.8)	30.0 (5.0, 284.0)	0.75 (0.38, 11.08), *p* = 0.41	48.0 (2.7, 256.2)	0.65 (0.10, 4.02), *p* = 0.33
EpCAM + ^b^	9.0 (2.0, 26.0)	15.0 (8.5, 38.5)	5.17 (0.76, 18.3), *p* = 0.037	19.0 (3.0, 39.3)	1.05 (0.21, 3.59), *p* = 0.15
ALDH + EpCAM + ^c^	2.0 (1.0, 14.0)	5.0 (2.0, 13.5)	1.0 (3.0, 7.0), *p* = 0.53	3.0 (1.0, 25.5)	1.0 (0.05, 2.69), *p* = 0.17

^a^Of those with samples, circulating tumour measurements of ALDH were missing for three individuals at pre-treatment, one individual post chemotherapy and two individuals post vismodegib. Ratio change was missing for one patient after chemotherapy, and three patients after vismodegib

^b^Of those with samples, circulating tumour measurements of EpCAM were missing for four individuals at pre-treatment, one individual post chemotherapy and two individuals post vismodegib. Ratio change was missing for one patient after chemotherapy, and four patients after vismodegib

^c^Of those with samples, circulating tumour measurements of ALDH + EpCAM + were missing for five individuals at pre-treatment, one individual post chemotherapy and seven individuals post vismodegib. Fold change was missing for two patients after chemotherapy, and ten patients after vismodegib

The median (1st quartile, 3rd quartile) provided for each measurement

#### IHC staining for GLI-1

IHC Gli-1 assessment was available for 49 pre-treatment biopsies. Of these, 31 had matching post-treatment biopsy (11 after receiving chemotherapy only [gemcitabine and nab-paclitaxel without the effect of hedgehog inhibitor], 20 had matching post-Hh vismodegib samples) and 18 did not have matching follow-up GLI-1 data. Of the paired measurements, only three changed from negative staining to positive staining during follow-up. Baseline measurements of GLI-1 were not significantly associated with PFS or OS (*p* > 0.05, Supplementary Tables 1, 2).

IHC GLI-1 assessment in the stromal compartment was evaluable for 23 paired biopsies. Only four had a noted change in staining during follow-up.

#### Tumour differentiation

IHC assessment of tumour differentiation was assessed in H&E samples, and available for 49 pre-treatment biopsies and 31 paired post-treatment biopsies. The majority (*N* = 18) remained with the same tumour differentiation, while nine changed to worse differentiation and four changed for the better.

#### RT-PCR for Gli-1 and PATCH

**Fig. 2:**
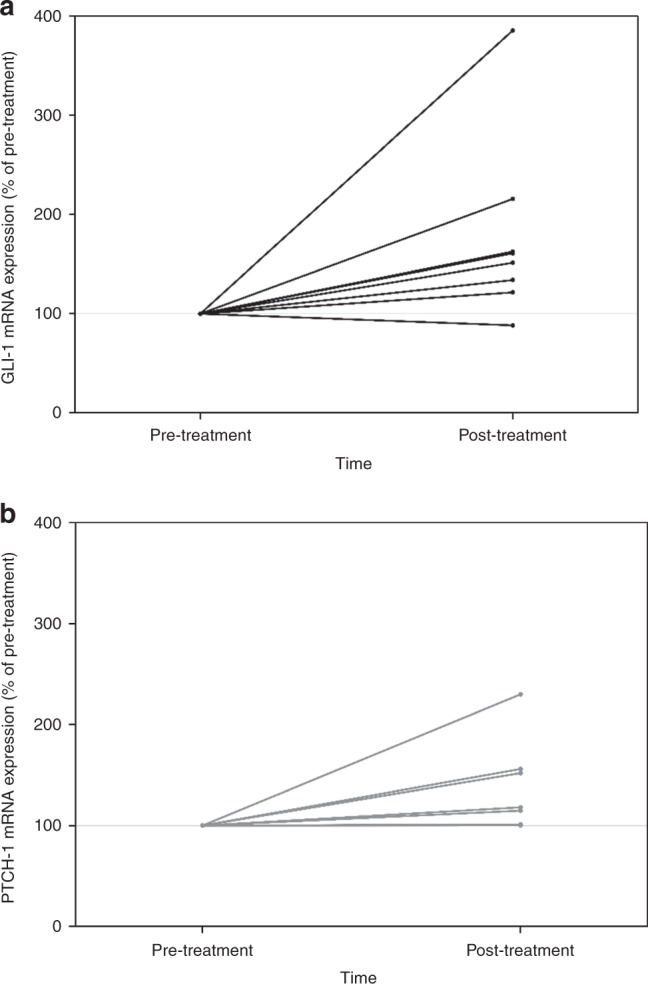
RT-PCR for GLI-1 and PATCH comparing baseline pre-treatment and post- treatment expression. The post-treatment samples were collected following exposure to vismodegib therapy. **a** GLI-1. **b** PATCH.

We had limited samples with adequate RNA to measure Gli-1 and PATCH expression using RT-PCR (*n* = 8, Fig. 2) largely because of limited tumour tissue. There was a significant increase from baseline in both GLI-1 (*p* = 0.016) and PTCH-1 (*p* = 0.008) from pre- to post-vismodegib treatment measurements. Baseline measurements for GLI were median 5.2 (range 1.2–7.0) and PTCH was 3.5 (range 1.4–6.5).

#### Immunofluorescence staining for stromal markers and immune infiltrates

We had a total of nine paired biopsies after administration of chemotherapy only. We had a total of 13 paired biopsies after administration of Hh inhibitor vismodegib. There was no statistically significant change from baseline epithelial (E-cadherin), stromal (SMA) or immune (CD45 + ) expression with chemotherapy or vismodegib (*p* > 0.05). Higher levels of SMA at baseline (≥ 5.37%) were associated with significantly worse PFS (HR: 2.44, 95% CI: 1.21–4.93, *p* = 0.01); however, there was no significant difference in OS or response for those with high vs. low SMA at baseline. Baseline levels of E-cadherin and CD45 + were not significantly associated with PFS, OS or response (*p* > 0.05). Correction for multiple comparisons was not performed.

## Discussion

Targeting the Hh signalling pathway in patients with PDA was of interest after preclinical studies, suggesting that Hh signalling inhibition could deplete pCSC, the stromal compartment, and result in increased drug delivery.^12,19^ This clinical trial was designed to assess the safety and clinical benefit of one such therapy, vismodegib, after extensive preclinical evidence, suggesting benefit when combining Hh inhibitors with chemotherapy.^8,10,19,26–29^ Our approach of an initial cycle chemotherapy prior to administration of vismodegib starting on cycle 2 of chemotherapy was based on one of these preclinical studies that showed sequencing of chemotherapy first (gemcitabine) to deplete the bulk of tumour cells followed by the administration of a Hh inhibitor decreased the number of chemotherapy-resistant pCSC.^30^

The efficacy of the new treatment combination, measured by the primary outcome PFS, was compared with a historical control of standard chemotherapy with gemcitabine and nab-paclitaxel. A Phase 3 trial of gemcitabine and nab-paclitaxel reported a PFS of 5.5 months and an OS of 8.5 months.^3^ We observed similar results with a PFS of 5.42 months and an OS of 9.79 months, respectively. Therefore, the primary endpoint of increasing PFS was not met. Several clinical trials testing Hh inhibition with chemotherapy occurred parallel to our study. A randomised, Phase 2 clinical trial comparing gemcitabine combined with the Hh inhibitor IPI-926 (saridegib) vs. gemcitabine with placebo was closed early after an interim analysis that found a significantly reduced PFS and OS in the group of patients treated with the combination of gemcitabine plus the Hh inhibitor.^31^ Similar to our results, a randomised Phase 2 study comparing gemcitabine with Hh inhibitor vismodegib vs. gemcitabine alone failed to demonstrate a benefit or detriment in PFS.^32^ They used only gemcitabine as the chemotherapy backbone, and our study used a more efficacious combination of gemcitabine with nab-paclitaxel. Despite the combination, there was no improvement in efficacy by adding vismodegib therapy. We did not identify new AEs related to study drugs. Some of the AEs known to occur with single agent vismodegib were observed at a higher frequency in our trial (Supplementary Table 4).^20^ However, the majority of these are overlapping toxicities also known to occur with the administration of chemotherapy agents or due to the underlying disease.

We acknowledge the limitations for survival assessment. Firstly, we lacked the benefits of a randomised concurrent control to assess for either an improvement or detrimental change in survival. Secondly, once study initiated the design was modified to use as a benchmark of survival the data from the results of the Phase 3 IMPACT trial instead of the initial historical results from the Phase 1–2 trial with gemcitabine and nab-paclitaxel. Our rationale for this change was to ensure that we treated only the appropriate number of patients to answer the clinical question of improvement of PFS. We acknowledge that approach can potentially lead to overemphasising single-arm Phase 2 trial results, which traditionally report better efficacy results as compared with global Phase 3 studies, usually due to highly selected patients and academic centres with large expertise. Every effort was made to avoid patient selection, and the primary endpoint PFS with its radiographic responses was determined by independent radiology review.

We analysed multiple biomarkers. Most prognostic risk factors, including the standard cut-off for CA 19-9, were not associated with PFS or OS. A very high CA 19-9 at baseline (> 5000) was associated with shorter time to progression and death. Further investigation and validation of the alternate threshold for CA 19-9 as a prognostic biomarker are warranted. The results of our correlative studies are exploratory but allowed us to generate hypothesis for lack of efficacy.

### Hh signalling is upregulated despite vismodegib and chemotherapy

When using IHC, we observed no significant changes in the expression of GLI-1. We also analysed GLI-1 mRNA expression using RT-PCR in a subset of patients with adequate RNA in their paired pre–post-treatment samples after vismodegib exposure. Although our sample size was limited (*n* = 8), we observed a statistically significant upregulation of Hh signalling at the mRNA translation level (Fig. 2). As RT-PCR can detect changes earlier than IHC staining, this suggests Hh signalling activation despite SMO inhibition. Kim et al. conducted a clinical trial in which patients were treated with vismodegib followed by combined vismodegib with gemcitabine.^33^ They reported a decrease in Hh signalling as measured by Gli-1 and PTCH mRNA levels in their paired biopsies. In their study, the investigators obtained the post-treatment sample after 3 weeks of administration of vismodegib monotherapy in the absence of chemotherapy. We believe that this may potentially explain the discrepancy with our results and hypothesised that there is a chemotherapy-induced mechanism that results in Hh signalling activation despite Hh inhibitor administration. To date, Hh inhibitors have been shown beneficial when used as single-agent therapy in basal cell carcinoma,^30,34–36^ and studies that have combined these agents with chemotherapy have failed to show clinical benefit.^37^ Similar to our findings, there was no correlation between baseline Gli-1 expression and survival.

### pCSC expression remained unchanged despite chemotherapy and Hh inhibition

We used ALDH IHC as a marker for pCSC.^14^ We observed few changes in IHC staining for ALDH after administration of chemotherapy combined with vismodegib or chemotherapy only. Our findings support preclinical studies that described that pCSC are highly resistant to chemotherapy. However, contrary to preclinical reports, there were no changes in the IHC staining of ALDH and hence no changes in the pCSC compartment despite administration of Hh inhibition.^27,30^ Our findings suggest that the Hh pathway as the only molecular target for pCSC is not sufficient to deplete the stem cell compartment. We hypothesise that alternative pathways described in stem cell biology may be more clinically significant drivers of pCSC.

### It is possible to measure circulating pCSC in patients with PDA while on treatment

Circulating tumour cells (CTCs) with tumour-initiating phenotype properties including ALDH can be detected in peripheral blood and when present are independently predictive of decreased disease-free and overall survival.^38^ We utilised flow cytometry to detect CTCs based on expression of EpCAM and lack of expression of CD45 and CD31. A subset of these CTCs was characterised as pCSCs based on high ALDH activity as measured using the ALDEFLUOR assay. Measuring circulating pCSC could be a non-invasive technique in future studies that seek to correlate changes of stem cell biology in response to treatments. In our study, baseline elevation of pCSC did not impact clinical outcomes.

### Hh inhibitors did not cause a change in stromal component

We used actin SMA as a marker of myofibroblasts, the main component of the PDA desmoplastic stroma.^21^ We did not observe an effect of stromal component when analysing paired pre–post-treatment biopsies. This finding differs from previous studies that suggested a decrease in stromal compartment after Hh inhibition.^21^ Our samples were obtained after one cycle following Hh inhibition, and it is possible that the duration of therapy was not sufficient to observe a decline. Interestingly, higher levels of SMA at baseline (≥ 5.37%) were associated with significantly worse PFS although there was no significant difference in OS or response to treatment for those with high vs. low SMA. A previous study reported that high stromal activity, representing activated pancreatic stellate cells in PDAs, was not significantly associated with the prognosis of resected PDAs.^39^ Our findings were primarily exploratory and hypothesis generating; thus, further investigation and validation of SMA as a prognostic biomarker in trials targeting the stromal compartment are warranted.

### It is challenging to obtain paired tumour biopsies in patients with PDA

Baseline biopsies are feasible but there is a significant dropout rate for obtaining the second paired tissue specimen (secondary to progression and/or inaccessible tumours). There are technical challenges with small core biopsies as these would limit the amount of tumour tissue for adequate IHC. We used a small 22-gauge needle to minimise the risk. There were no complications while obtaining the biopsies, but the small size of the cores limited the amount of tissue for adequate IHC staining or RNA extraction. Future studies using correlatives are important. With the challenges we had in this study, a suggestion would be to obtain larger core biopsies and planning the biopsies as close to study enrollment as possible. In the case of mandatory biopsies, ~72% of the patients enrolled had a biopsy with adequate tissue for correlative studies at baseline (prior to initiating therapy), but there was a significant reduction with 69% having a second biopsy within 1–2 months from enrolment.

## Conclusions

Adding the Hh inhibitor vismodegib to gemcitabine and nab-paclitaxel did not improve efficacy as compared with historical rates observed with chemotherapy alone in patients with newly diagnosed metastatic pancreatic cancer. This study does not support the further evaluation of Hh inhibitors in this patient population.

## Supplementary information

Supplementary files combined

## Data Availability

The data are available at Johns Hopkins’ secured system and as needed per request.
