# Supplementary material for: Phase 2 study of vismodegib, a hedgehog inhibitor, combined with gemcitabine and nab-paclitaxel in patients with untreated metastatic pancreatic adenocarcinoma
Source: Br J Cancer. 2019 Dec 20;122(4):498–505. doi: 10.1038/s41416-019-0683-3 (PMC7029016; doi:10.1038/s41416-019-0683-3)

**Supplementary Files**

**Figures and Tables Legends**

**Supplemental Figure 1** Consort diagram for clinical trial enrollment.

**Supplemental Table 1:** Risk factors for progression free survival (PFS).

**Supplemental Table 2:** Risk factors for overall survival (OS).

**Supplemental Table 3:** Association between demographic and disease characteristics and response within 4 cycles.

**Supplemental Table 4:** Comparison of toxicities known to occur with vismodegib versus its frequency observed in the study.

**Supplemental Figure 2:** Flowchart of biopsies obtained

**Supplemental Figure 1:** Consort diagram for clinical trial enrollment.


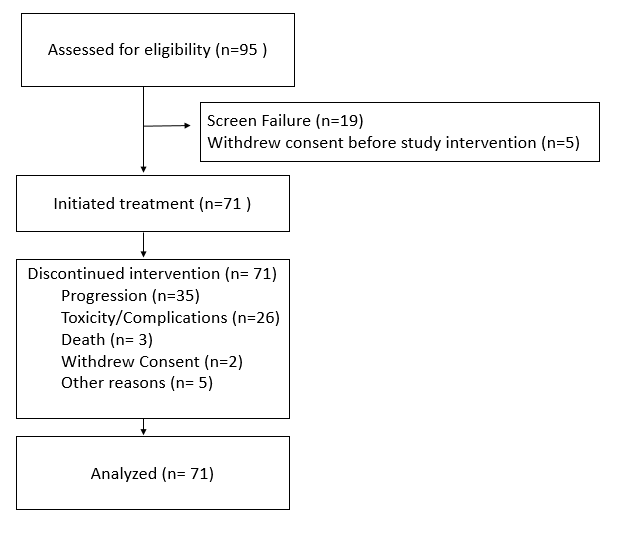


Supplemental Table 1. Risk factors for progression free survival (PFS).

| **Characteristic** | **# at risk** | **# events** | **Median**  **(95% CI)** | **Percent Progression Free at 6 months**  **(95% CI)** | **Hazard Ratio**  **(95% CI)** | **P-value** |
| --- | --- | --- | --- | --- | --- | --- |
| Age |  |  |  |  |  |  |
| > 65 | 40 | 38 | 4.83 (3.61, 6.83) | 37.9% (25.2%, 56.9%) | 1.00 | 0.17 |
| ≤ 65 | 31 | 30 | 5.75 (4.34, 8.90) | 48.4% (33.6%, 69.7%) | 0.71 (0.43, 1.16) |  |
| Gender |  |  |  |  |  |  |
| Female | 34 | 33 | 5.22 (3.68, 7.33) | 42.5% (28.6%, 63.3%) | 1.00 | 0.91 |
| Male | 37 | 35 | 5.52 (4.24, 7.16) | 42.6% (29.1%, 62.2%) | 1.03 (0.63, 1.68) |  |
| Race |  |  |  |  |  |  |
| White | 63 | 61 | 5.52 (4.56, 7.13) | 43.2% (32.4%, 57.6%) | 1.00 |  |
| Black | 4 | 3 | 1.38 (0.62, Inf) | 25.0% (4.5%, 100%) | 0.84 (0.25, 2.78) | 0.78 |
| Asian | 3 | 3 | 3.91 (1.57, Inf) | 33.3% (6.7%, 100%) | 0.92 (0.28, 3.00) | 0.90 |
| Prior resection |  |  |  |  |  |  |
| No | 62 | 60 | 5.29 (4.34, 6.97) | 40.6% (29.9%, 55.2%) | 1.00 | 0.16 |
| Yes | 7 | 6 | 7.72 (1.45, 18.63) | 57.1% (30.0%, 100.0%) | 0.54 (0.22, 1.28) |  |
| ECOG status |  |  |  |  |  |  |
| 0 | 30 | 29 | 5.52 (3.68, 7.89) | 43.3% (28.7%, 65.3%) | 1.00 | 0.44 |
| 1 | 41 | 39 | 5.22 (4.17, 7.16) | 42.1% (29.1%, 60.8%) | 1.21 (0.74, 1.98) |  |
| Nodal status |  |  |  |  |  |  |
| Negative | 10 | 10 | 6.44 (1.84, 8.38) | 50.0% (26.9%, 93.0%) | 1.00 |  |
| Positive | 40 | 38 | 5.42 (3.19, 7.33) | 43.1% (29.9%, 62.1%) | 1.06 (0.52, 2.13) | 0.88 |
| Missing | 21 | 20 | 5.06 (4.17, 7.85) | 38.1% (22%, 65.8%) | 1.07 (0.49, 2.29) | 0.87 |
| CA 19-9 |  |  |  |  |  |  |
| Not elevated (< 37) | 10 | 9 | 7.00 (1.58, 10.58) | 50.0% (26.9%, 93.0%) | 1.00 | 0.089 |
| Elevated (≥ 37) | 53 | 52 | 5.52 (4.37, 6.97) | 43.0% (31.4%, 58.8%) | 1.93 (0.9, 4.15) |  |
|  |  |  |  |  |  |  |
| < 5000 | 44 | 42 | 6.97 (5.42, 8.38) | 56.4% (43.4%, 73.3%) | 1.00 | 0.0005 |
| ≥ 5000 | 19 | 19 | 4.14 (2.37, 5.22) | 15.8% (5.5%, 44.7%) | 2.80 (1.56, 5.03) |  |
|  |  |  |  |  |  |  |
| Biopsies |  |  |  |  |  |  |
| ALDH |  |  |  |  |  |  |
| No staining | 28 | 28 | 5.26 (3.68, 6.97) | 35.7% (21.7%, 58.8%) | 1.00 |  |
| Citoplasmic positive | 8 | 6 | 5.68 (0.92, ∞) | 19.4% (3.4%, 100%) | 0.89 (0.36, 2.2) | 0.81 |
| Both nuclear and  citoplasmic positive | 4 | 4 | 8.33 (4.86, ∞) | 75% (42.5%, 100%) | 0.68 (0.23, 1.96) | 0.47 |
| GLI-1 |  |  |  |  |  |  |
| No staining | 9 | 9 | 2.37 (0.657, 9.92) | 22.2% (6.5%, 75.5%) | 1.00 |  |
| Citoplasmic positive | 18 | 17 | 5.36 (3.91, 7.13) | 33.3% (17.3%, 64.1%) | 0.76 (0.33, 1.73) | 0.51 |
| Both nuclear and  citoplasmic positive | 22 | 20 | 6.24 (4.337, 8.77) | 51.3% (33.5%, 78.4%) | 0.71 (0.32, 1.58) | 0.40 |
| Stroma |  |  |  |  |  |  |
| No staining | 15 | 15 | 5.52 (1.15, 8.38) | 33.3% (16.2%, 68.2%) | 1.00 |  |
| Citoplasmic positive | 15 | 14 | 4.86 (2.99, 6.24) | 33.3% (16.2%, 68.2%) | 1.13 (0.54, 2.36) | 0.75 |
| Both nuclear and  citoplasmic positive | 11 | 11 | 7.16 (3.68, 9.66) | 54.5% (31.8%, 93.6%) | 0.96 (0.43, 2.13) | 0.93 |
| Tumor differentiation |  |  |  |  |  |  |
| Poor | 15 | 13 | 5.29 (1.58, 7.89) | 35.9% (17.8%, 72.4%) | 1.00 |  |
| Moderate | 7 | 7 | 5.68 (0.92, 14.92) | 28.6% (8.8%, 92.2%) | 0.74 (0.29, 1.89) | 0.53 |
| Well | 26 | 25 | 5.22 (3.91, 7.16) | 41.4% (26%, 65.9%) | 0.87 (0.43, 1.72) | 0.68 |
|  |  |  |  |  |  |  |
| Circulating Tumor Cells* | | | | | | |
| ALDH+ |  |  |  |  |  |  |
| < 27 | 27 | 25 | 4.57 (2.99, 6.97) | 36.7% (22.2%, 60.5%) | 1.00 | 0.47 |
| ≥ 27 | 27 | 26 | 6.24 (3.68, 8.38) | 50.1% (34.1%, 73.6%) | 0.81 (0.46, 1.42) |  |
|  |  |  |  |  |  |  |
| EpCAM+ |  |  |  |  |  |  |
| < 7 | 26 | 24 | 4.34 (2.53, 6.97) | 38.5% (23.6%, 62.6%) | 1.00 | 0.61 |
| ≥ 7 | 27 | 27 | 5.75 (4.24, 8.38) | 48.1% (32.5%, 71.3%) | 0.86 (0.49, 1.51) |  |
|  |  |  |  |  |  |  |
| ALDH+ EpCAM+ |  |  |  |  |  |  |
| < 2 | 23 | 21 | 4.83 (3.91, 7.33) | 43.0% (26.7%, 69.2%) | 1.00 | 0.78 |
| ≥ 2 | 29 | 28 | 5.75 (3.68, 8.38) | 43.1% (28.1%, 66%) | 0.92 (0.51, 1.64) |  |
|  |  |  |  |  |  |  |
| Immunofluorescence* | | | | | | |
| e-cadherin |  |  |  |  |  |  |
| < 4.39% | 19 | 19 | 4.34 (2.2, 6.97) | 31.6% (16.2%, 61.3%) | 1.00 | 0.63 |
| ≥ 4.39% | 20 | 19 | 5.29 (4.24, 7.89) | 36.9% (20.5%, 66.5%) | 0.85 (0.43, 1.65) |  |
|  |  |  |  |  |  |  |
| SMA |  |  |  |  |  |  |
| < 5.37% | 23 | 22 | 5.75 (4.24, 9.49) | 50.0% (32.9%, 76%) | 1.00 | 0.01 |
| ≥ 5.37% | 17 | 17 | 4.37 (1.84, 5.42) | 17.6% (6.3%, 49.3%) | 2.44 (1.21, 4.93) |  |
|  |  |  |  |  |  |  |
| CD45+ infiltration |  |  |  |  |  |  |
| < 3.25% | 20 | 20 | 5.22 (2.37, 5.75) | 25.0% (11.7%, 53.5%) | 1.00 | 0.70 |
| ≥ 3.25% | 19 | 18 | 4.85 (4.17, 7.72) | 44.4% (26.5%, 74.5%) | 1.14 (0.57, 2.27) |  |

CI = confidence interval.

* Medians were used as the thresholds for circulating tumor cells and immunofluorescence.

Supplemental Table 2: Risk factors for overall survival (OS).

| **Characteristic** | **# at risk** | **# events** | **Median**  **(95% CI)** | **Percent Alive at 6 months**  **(95% CI)** | **Hazard Ratio**  **(95% CI)** | **P-value** |
| --- | --- | --- | --- | --- | --- | --- |
| Age |  |  |  |  |  |  |
| > 65 | 40 | 38 | 9.80 (7.82, 11.20) | 80.0% (68.5%, 93.5%) | 1.00 | 0.78 |
| ≤ 65 | 31 | 26 | 9.80 (5.42, 12.00) | 67.7% (53.1%, 86.4%) | 0.93 (0.55, 1.55) |  |
| Gender |  |  |  |  |  |  |
| Female | 34 | 30 | 9.45 (5.42, 13.90) | 64.7% (50.4%, 83.0%) | 1.00 | 0.78 |
| Male | 37 | 34 | 9.79 (7.85, 11.80) | 83.8% (72.7%, 96.6%) | 1.07 (0.65, 1.77) |  |
| Race |  |  |  |  |  |  |
| White | 63 | 57 | 9.79 (7.85, 11.00) | 76.2% (66.3%, 87.5%) | 1.00 |  |
| Black | 4 | 3 | 9.68 (0.62, Inf) | 75.0% (42.5%, 100%) | 0.94 (0.29, 3.01) | 0.91 |
| Asian | 3 | 3 | 5.13 (3.91, Inf) | 33.3% (6.7%, 100%) | 1.53 (0.47, 4.93) | 0.48 |
| Prior resection |  |  |  |  |  |  |
| No | 62 | 56 | 9.56 (7.82, 10.7) | 75.8% (65.8%, 87.3%) | 1.00 | 0.27 |
| Yes | 7 | 6 | 13.9 (1.45, Inf) | 71.4% (44.7%, 100%) | 0.62 (0.26, 1.46) |  |
| ECOG status |  |  |  |  |  |  |
| 0 | 30 | 24 | 10.20 (7.89, 14.30) | 80.0% (66.8%, 95.7%) | 1.00 | 0.09 |
| 1 | 41 | 40 | 9.10 (6.11, 10.70) | 70.7% (58.0%, 86.2%) | 1.56 (0.93, 2.61) |  |
| Nodal status |  |  |  |  |  |  |
| Negative | 10 | 10 | 10.10 (3.52, 15.80) | 80.0% (58.6%, 100%) | 1.00 |  |
| Positive | 40 | 35 | 10.20 (7.82, 11.20) | 75.0% (62.7%, 89.7%) | 0.90 (0.44, 1.83) | 0.77 |
| Missing | 21 | 19 | 9.10 (5.59, 14.30) | 71.4% (54.4%, 93.7%) | 0.97 (0.44, 2.11) | 0.94 |
| CA 19-9 |  |  |  |  |  |  |
| Not elevated (< 37) | 10 | 7 | 12.3 (5.1, Inf) | 80.0% (58.6%, 100%) | 1.00 | 0.24 |
| Elevated (≥ 37) | 53 | 40 | 10.0 (7.8, 11.5) | 77.4% (66.8%, 89.5%) | 1.62 (0.72, 3.6) |  |
|  |  |  |  |  |  |  |
| < 5000 | 44 | 37 | 11.32 (9.79, 14.32) | 88.6% (79.7%, 98.6%) | 1.00 | 0.0001 |
| ≥ 5000 | 19 | 19 | 6.11 (4.14, 9.56) | 52.6% (34.3%, 80.7%) | 3.15 (1.74, 5.7) |  |
|  |  |  |  |  |  |  |
| Biopsies |  |  |  |  |  |  |
| ALDH |  |  |  |  |  |  |
| No staining | 28 | 27 | 7.26 (5.42, 9.1) | 67.9% (52.5%, 87.6%) | 1.00 |  |
| Citoplasmic positive | 8 | 8 | 9.71 (2.04, 10.7) | 87.5% (67.3%, 100%) | 0.95 (0.42, 2.13) | 0.90 |
| Both nuclear and  citoplasmic positive | 4 | 4 | 10.63 (4.86, NA) | 75% (42.5%, 100%) | 0.89 (0.3, 2.58) | 0.82 |
| GLI-1 |  |  |  |  |  |  |
| No staining | 9 | 9 | 7.29 (0.657, 17.3) | 55.6% (30.9%, 99.7%) | 1.00 |  |
| Citoplasmic positive | 18 | 15 | 8.59 (6.078, 13.9) | 77.8% (60.7%, 99.6%) | 0.76 (0.33, 1.76) | 0.52 |
| Both nuclear and  citoplasmic positive | 22 | 21 | 9.79 (7.261, 11) | 86.4% (73.1%, 100%) | 0.75 (0.34, 1.66) | 0.48 |
| Stroma |  |  |  |  |  |  |
| No staining | 15 | 15 | 7.85 (3.38, 9.99) | 66.7% (46.6%, 95.4%) | 1.00 |  |
| Citoplasmic positive | 15 | 14 | 7.26 (4.86, 10.97) | 73.3% (54%, 99.6%) | 1.02 (0.48, 2.16) | 0.95 |
| Both nuclear and  citoplasmic positive | 11 | 10 | 9.79 (5.36, 20.63) | 81.8% (61.9%, 100%) | 0.83 (0.36, 1.9) | 0.66 |
| Tumor differentiation |  |  |  |  |  |  |
| Poor | 15 | 12 | 8.18 (4.34, 10.5) | 73.3% (54%, 99.6%) | 1.00 |  |
| Moderate | 7 | 7 | 9.10 (3.52, 20.6) | 85.7% (63.3%, 100%) | 1.05 (0.40, 2.69) | 0.92 |
| Well | 26 | 25 | 9.89 (6.31, 11.8) | 76.9% (62.3%, 95%) | 1.01 (0.50, 2.03) | 0.98 |
|  |  |  |  |  |  |  |
| Circulating Tumor Cells* | | | | | | |
| ALDH+ |  |  |  |  |  |  |
| < 27 | 27 | 23 | 8.08 (4.14, 11.5) | 55.6% (39.6%, 77.9%) | 1.00 | 0.81 |
| ≥ 27 | 27 | 26 | 9.79 (9.10, 13.7) | 92.6% (83.2%, 100%) | 0.93 (0.52, 1.66) |  |
|  |  |  |  |  |  |  |
| EpCAM+ |  |  |  |  |  |  |
| < 7 | 26 | 23 | 10.1 (5.39, 11.5) | 65.4% (49.4%, 86.5%) | 1.00 | 0.85 |
| ≥ 7 | 27 | 25 | 9.8 (7.82, 14.3) | 81.5% (68%, 97.6%) | 0.95 (0.53, 1.69) |  |
|  |  |  |  |  |  |  |
| ALDH+ EpCAM+ |  |  |  |  |  |  |
| < 2 | 23 | 18 | 10.71 (5.39, 17.6) | 65.2% (48.3%, 87.9%) | 1.00 | 0.14 |
| ≥ 2 | 29 | 29 | 9.56 (6.9, 10.2) | 79.3% (65.8%, 95.6%) | 1.59 (0.86, 2.91) |  |
|  |  |  |  |  |  |  |
| Immunofluorescence* | | | | | | |
| e-cadherin |  |  |  |  |  |  |
| < 4.39% | 19 | 18 | 7.82 (4.34, 10.51) | 63.2% (44.8%, 89.1%) | 1.00 | 0.78 |
| ≥ 4.39% | 20 | 20 | 8.18 (5.42, 9.99) | 75% (58.2%, 96.6%) | 0.91 (0.47, 1.76) |  |
|  |  |  |  |  |  |  |
| SMA |  |  |  |  |  |  |
| < 5.37% | 23 | 23 | 9.79 (7.82, 10.5) | 78.3% (63%, 97.1%) | 1.00 | 0.29 |
| ≥ 5.37% | 17 | 16 | 6.11 (5.13, 9.8) | 58.8% (39.5%, 87.6%) | 1.43 (0.74, 2.76) |  |
|  |  |  |  |  |  |  |
| CD45+ infiltration |  |  |  |  |  |  |
| < 3.25% | 20 | 20 | 7.82 (5.13, 10.5) | 60% (41.9%, 85.9%) | 1.00 | 0.93 |
| ≥ 3.25% | 19 | 18 | 9.49 (6.08, 10.3) | 78.9% (62.5%, 99.6%) | 0.97 (0.5, 1.87) |  |

CI = confidence interval.

* Medians were used as the thresholds for circulating tumor cells and immunofluorescence.

Supplemental Table 3. Association between demographic and disease characteristics and response within 4 cycles.

| **Characteristic*** | **No Response**  **within 4 cycles**  **(N = 42)** | **Response within 4 cycles**  **(N = 25)** | **P-value**† |
| --- | --- | --- | --- |
| *Demographics* |  |  |  |
| Age at enrollment, median (Q1-Q3) | 60 (56 – 69) | 65 (57-70) | 0.71 |
| Male, N(%) | 21 (50%) | 15 (60%) | 0.46 |
| Race  White  Black  Asian | 36 (88%)  3 (7%)  2 (5%) | 23 (92%)  1 (4%)  1 (4%) | > 0.99 |
| *Disease status* |  |  |  |
| ECOG at enrollment, N(%)  0  1 | 19 (45%)  23 (55%) | 10 (40%)  15 (60%) | 0.80 |
| Current disease involvement, N(%)  Primary tumor  Liver  Regional lymph nodes  Lung  Distant lymph nodes  Peritoneum | 36 (88%)  36 (88%)  17 (41%)  10 (24%)  10 (24%)  8 (20%) | 20 (83%)  20 (83%)  8 (33%)  6 (25%)  4 (17%)  4 (17%) | 0.72  0.72  0.60  > 0.99  0.55  > 0.99 |
| Nodal status, N(%)  Positive  Negative  Missing | 24 (57%)  6 (14%)  12 (29%) | 13 (52%)  4 (16%)  8 (32%) | 0.94 |
| Prior surgical resection, N(%) | 4 (10%) | 2 (8%) | > 0.99 |
| CA19-9 at enrollment  Median (Q1-Q3)  Elevated (≥ 37), N(%)  ≥ 5000, N(%) | 1392 (126-8825)  33 (82%)  14 (35%) | 1509 (120-3847)  19 (86%)  5 (23%) | 0.59  > 0.99  0.40 |
| *Biopsies, N(%)* |  |  |  |
| ALDH  Negative  Citoplasmic staining positive  Both nuclear and citoplasmic  staining positive | 16 (70%)  5 (22%)  2 (9%) | 10 (71%)  2 (14%)  2 (14%) | 0.88 |
| GLI-1  Negative  Citoplasmic staining positive  Both nuclear and citoplasmic  staining positive | 5 (19%)  11 (42%)  10 (38%) | 3 (15%)  6 (30%)  11 (55%) | 0.62 |
| Stroma  Negative  Citoplasmic staining positive  Both nuclear and citoplasmic  staining positive | 10 (48%)  5 (24%)  6 (29%) | 4 (22%)  9 (50%)  5 (28%) | 0.16 |
| Tumor differentiation  Poor  Moderate  Well | 8 (32%)  4 (16%)  13 (52%) | 5 (25%)  3 (15%)  12 (60%) | 0.92 |
| *Circulating Tumor Cells* |  |  |  |
| ALDH+, median (Q1-Q3) | 10 (2-169) | 59 (10-156) | 0.24 |
| EPCAM+, median (Q1-Q3) | 10 (2-38) | 7 (1-26) | 0.24 |
| ALDH+EPCAM+, median (Q1-Q3) | 2 (1-16) | 2 (1-13) | 0.96 |
| *Immunofluorescence staining (%)* |  |  |  |
| e-cadherin, median (Q1-Q3) | 6.9 (0.5-25.0) | 3.4 (0-44.7) | 0.96 |
| Smooth Muscle Actin | 5.5 (3.5-7.2) | 5.1 (2.3-7.7) | 0.54 |
| CD45+ cells infiltration | 3.0 (1.4-5.7) | 3.3 (0.9-6.3) | 0.91 |

* Missing data: race (N = 1), current disease involvement (N = 2), nodal status (N = 20), prior resection (N = 2), CA 19-9 (N = 2), baseline tumor assessments (ALDH: N = 30, GLI: N = 21, stroma: N = 28, tumor differentiation: N = 22), baseline circulating tumor cells (ALDH+: N = 16, EPCAM+ : N = 16, ALDH+EPCAM+: N = 18).

† P-values are based upon Fisher’s exact tests and Wilcoxon rank sum tests.

Q1 = 1^st^ quartile; Q3 = 3^rd^ quartile; N = number; % = percent.

**Supplemental Table 4: Comparison of toxicities known to occur with vismodegib versus its frequency observed in the study**

| AE Type | Current Study | | | | | Former Study (vismodegib only) | | | | |
| --- | --- | --- | --- | --- | --- | --- | --- | --- | --- | --- |
|  | AE Grade | | | | | AE Grade | | | | |
|  | 1 | 2 | 3 | 4 | Total | 1 | 2 | 3 | 4 | Total |
| **Muscle spasms/**  **Myalgias** | 8 (11%) | 1 (1%) | 0 | 0 | 9 (13%) | 26 (38%) | 5  (7%) | 1 (2%) | 0 | 32 (47%) |
| **Dysgeusia** | 32 (45%) | 20 (28%) | 1 (1%) | 0 | 53 (75%) | 25 (37%) | 3  (4%) | 0 | 0 | 38 (41%) |
| **Fatigue** | 19 (27%) | 24 (34%) | 6 (8%) | 0 | 49 (69%) | 15 (22%) | 8 (12%) | 4 (6%) | 1 (2%) | 28 (41%) |
| **Alopecia** | 28 (39%) | 18 (25%) | 0 | 0 | 46 (65%) | 22 (32%) | 2  (3%) | 0 | 0 | 24 (35%) |
| **Nausea** | 26 (37%) | 12 (17%) | 3 (4%) | 0 | 41 (58%) | 18 (27%) | 4  (6%) | 1 (2%) | 0 | 23 (34%) |
| **Decreased appetite** | 13 (18%) | 12 (17%) | 1 (1%) | 0 | 26 (37%) | 15 (22%) | 4  (6%) | 0 | 0 | 19 (28%) |
| **Diarrhea** | 21 (30%) | 5 (7%) | 4 (6%) | 0 | 30 (42%) | 14 (21%) | 4  (6%) | 0 | 0 | 18 (27%) |
| **Weight loss** | 8 (11%) | 6 (8%) | 2 (3%) | 0 | 16 (23%) | 11 (16%) | 4  (6%) | 3 (4%) | 0 | 18 (27%) |
| **Cough** | 13 (18%) | 3 (4%) | 1 (1%) | 0 | 17 (24%) | 13 (19%) | 2  (3%) | 0 | 0 | 15 (22%) |
| **Back pain** | 4 (6%) | 1 (1%) | 1 (1%) | 0 | 6 (8%) | 3 (12%) | 3  (4%) | 2 (3%) | 0 | 13 (19%) |
| **Dyspnea** | 16 (23%) | 4 (6%) | 3 (4%) | 0 | 23 (32%) | 6 (9%) | 4  (6%) | 3 (4%) | 0 | 13 (19%) |
| **Constipation** | 12 (17%) | 5 (7%) | 0 | 0 | 17 (24%) | 9 (13%) | 3  (4%) | 0 | 0 | 12 (18%) |
| **Vomiting** | 21 (30%) | 6 (8%) | 3 (4%) | 0 | 30 (42%) | 8 (12%) | 3  (4%) | 1 (2%) | 0 | 12 (18%) |
| **Abdominal pain** | 9 (13%) | 8 (11%) | 2 (3%) | 0 | 19 (27%) | 3 (4%) | 3  (4%) | 5 (7%) | 0 | 11 (16%) |
| **Anxiety** | 7 (10%) | 0 | 0 | 0 | 7 (10%) | 7 (10%) | 3 (4%) | 0 | 0 | 10 (15%) |
| **Hyponatremia** | 12 (17%) | 1 (1%) | 10  (14%) | 1  (1%) | 24 (34%) | 1 (2%) | 1  (2%) | 7 (10%) | 1 (2%) | 10 (15%) |
| **Pruritus** | 12 (17%) | 0 | 0 | 0 | 12 (17%) | 9 (13%) | 0 | 0 | 0 | 9 (13%) |
| **Upper respiratory tract infection** | 1 (1%) | 2 (3%) | 0 | 0 | 3  (4%) | 7 (10%) | 2  (3%) | 0 | 0 | 9 (13%) |
| **Dyspepsia** | 2 (3%) | 0 | 0 | 0 | 2 (3%) | 7 (10%) | 1  (2%) | 0 | 0 | 8 (12%) |
| **Arthralgia** | 3 (4%) | 0 | 0 | 0 | 3 (4%) | 6 (9%) | 1  (2%) | 0 | 0 | 7 (10%) |
| **Hypokalemia** | 13 (18%) | 2 (3%) | 5 (7%) | 0 | 20 (28%) | 7 (10%) | 0 | 0 | 0 | 7 (10%) |
| **Hypomagnesemia** |  |  |  |  |  | 7 (10%) | 0 | 0 | 0 | 7 (10%) |
| **Pain*** | 13 (18%) | 9 (13%) | 3 (4%) | 0 | 25 (35%) | 5 (7%) | 0 | 2 (3%) | 0 | 7 (10%) |

**Supplemental Figure 2:** Flowchart of biopsies obtained


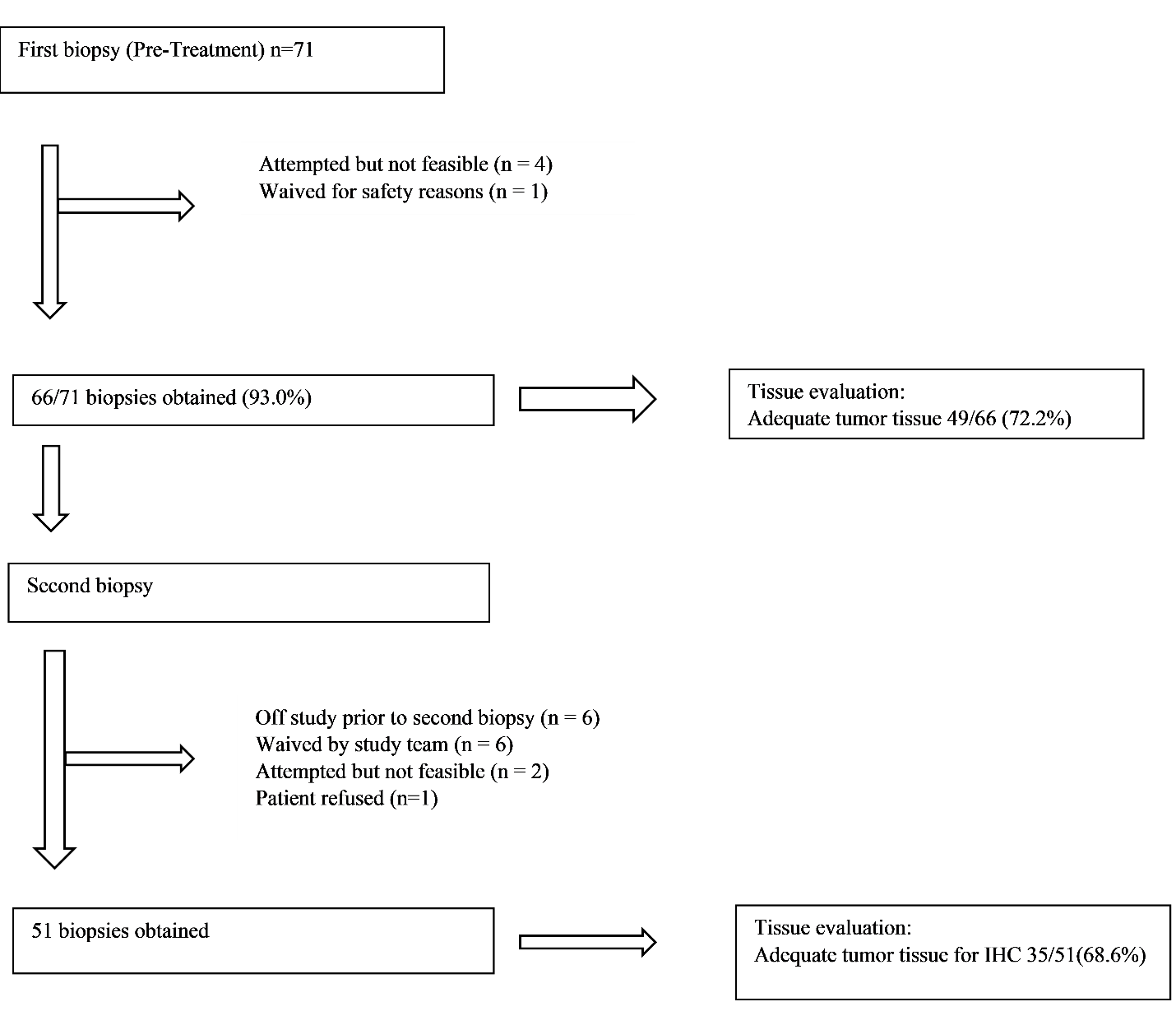

Supplement: Supplementary file 1 — Supplementary files combined [file 41416_2019_683_MOESM1_ESM.docx]
